# A grammar-based distance metric enables fast and accurate clustering of large sets of 16S sequences

**DOI:** 10.1186/1471-2105-11-601

**Published:** 2010-12-17

**Authors:** David J Russell, Samuel F Way, Andrew K Benson, Khalid Sayood

**Affiliations:** 1 Department of Electrical Engineering, University of Nebraska-Lincoln, 209N WSEC, Lincoln, NE, 68588-0511 USA; 2 Department of Food Science and Technology, University of Nebraska-Lincoln, 143 Filley Hall, Lincoln, NE, 68583-0919 USA; 3 Core for Applied Genomics and Ecology, University of Nebraska-Lincoln, 143 Filley Hall, Lincoln, NE, 68583-0919 USA

## Abstract

**Background:**

We propose a sequence clustering algorithm and compare the partition quality and execution time of the proposed algorithm with those of a popular existing algorithm. The proposed clustering algorithm uses a grammar-based distance metric to determine partitioning for a set of biological sequences. The algorithm performs clustering in which new sequences are compared with cluster-representative sequences to determine membership. If comparison fails to identify a suitable cluster, a new cluster is created.

**Results:**

The performance of the proposed algorithm is validated via comparison to the popular DNA/RNA sequence clustering approach, CD-HIT-EST, and to the recently developed algorithm, UCLUST, using two different sets of 16S rDNA sequences from 2,255 genera. The proposed algorithm maintains a comparable CPU execution time with that of CD-HIT-EST which is much slower than UCLUST, and has successfully generated clusters with higher statistical accuracy than both CD-HIT-EST and UCLUST. The validation results are especially striking for large datasets.

**Conclusions:**

We introduce a fast and accurate clustering algorithm that relies on a grammar-based sequence distance. Its statistical clustering quality is validated by clustering large datasets containing 16S rDNA sequences.

## Background

The amount of biological information being gathered is growing faster than the rate at which it can be analyzed. Data clustering, which compresses the problem space by reducing redundancy, is one viable tool for managing the explosive growth of data. In general, clustering algorithms are designed to operate on a large set of related values, eventually generating a smaller set of elements that represent groups of similar data points. A central data element may then be used as the sole representative of a group.

Significant clustering work relating to bioinformatics may be traced to the late 1990 s when methods for quick generation of nonredundant (NR) protein databases were developed. These combined identical or nearly identical protein sequences into single entries [1-3]. The primary benefits of these methods include faster searches of the NR protein databases and reduced statistical bias in the query results [1]. Similarly, computer programs such as those in ICAtools [4] were developed for compressing DNA databases by removing redundant sequences found via clustering resulting in faster database queries. Note that the use of the term "clustering" in these applications differs from another use often found in the literature where clustering refers to generating a phylogenetic distance matrix, such as in [5]. The operation of clustering used in this work identifies groups of sequences related by phylogeny; and it additionally applies to redundancy removal by identifying a sequence that suitably represents similar sequences.

Recently, DNA/RNA clustering has attracted attention for a variety of reasons. The drive to lower the expense of genome sequencing has led to the development of high-throughput sequencing technologies capable of generating millions of sequence fragments simultaneously. A clustering preprocessing step can be used to remove a great amount of fragment redundancy which, in turn, allows for quicker fragment reassembly.

One of the more popular DNA/RNA clustering algorithms is CD-HIT-EST [6] which was based on the protein clustering methods of [2,3] and was developed for clustering DNA/RNA database data such as non-intron-containing expressed sequence tags (ESTs).

A major application of CD-HIT has been for clustering large data sets from microbiota analysis (e.g. [7]), often as a preprocessing step to create sets of highly related sequences representing operational taxonomic units (OTUs). These OTUs are subsequently used as a basis for estimating species diversity between treatment groups or quantitative relationships of taxa between treatment groups. Alternatively, representative sequences from the OTUs are used for phylogeny-based analyses.

A recent effort in [8] to develop software tools which reduce the time required by BLAST [9] to search large biological databases has resulted in a set of programs, including UBLAST and USEARCH, that reduce the search time by orders of magnitude. As part of the work, an additional clustering program called UCLUST was created which utilizes the heuristic algorithm provided by USEARCH. UCLUST generates results that dramatically improve upon the time required by CD-HIT.

This work presents GramCluster, a fast and accurate algorithm for clustering large data sets of 16S rDNA sequences based on the inherent grammar of DNA and RNA sequences. Lempel-Ziv parsing [10] is used to estimate the grammar of each sequence to provide a distance metric among sequences. The implementation of this algorithm allows for fast and accurate clustering of biological information. The following sections describe the algorithm and present results, including comparisons with the CD-HIT-EST algorithm and the recently developed UCLUST algorithm.

## Results and Discussion

### Grammar

Necessary concepts for understanding how a grammar model is specified are briefly reviewed in this section. An *alphabet*, Σ, is a finite, nonempty set of symbols from which finite-length sequences, or *strings*, are formed. Strings are constructed via the binary operation of *concatenation *which begins with a copy of the left string and appends a copy of the right string. A *language*, *L*, is then defined as a subset of strings selected from the set of all strings over an alphabet, and a *problem *is defined as the question of deciding whether a given string is a member of some particular language. That is, given a string, *w *∈ Σ*, and *L*, a language over Σ, decide if *w *∈ *L*.

As *L *may be infinite, it is useful to have a compact description of the strings in *L*. Such an abstract model is called a *grammar*, *G*. Typically, a grammar is specified by the 4-tuple *G *= (*V*, *T*, *P*, *S*), where *V *is the set of *variables *and *T *is the set of *terminals *which are symbols that form the strings of *L*. *P *is the set of *productions*, each of which represent the recursive definition of *L*; and *S *∈ *V *is the *start symbol*, which is the variable that defines *L*. Each production consists of a *head *variable followed by the production operator → and a *body *string of zero or more terminals and variables. Each production represents one way to form strings in *L *from the head variable.

Given *G *= (*V*, *T*, *P*, *S*), the language, *L*, is defined by

$$L(G)=\{\;w|w\in T^{*},\text{ and }S\;\Rightarrow^{*}w\}.$$

That is, *L*(*G*) is the set of all strings derived from *S*.

It was observed in [11], that a grammar, *G*, used to model a string can be converted to an LZ77 representation in a simple way. The term LZ77 refers to Lempel-Ziv dictionary-based lossless compression detailed in [10] and [12]. Subsequently, an algorithm was presented in [13] to use an inverted process to map an LZ77-compressed sequence into a grammar. While the inverted process is more involved, it demonstrates the fact that Lempel-Ziv compression can be thought of as inferring a grammar from the sequence it compresses. The original concept behind abstract grammars is that a grammar, *G*, is meant to completely describe the underlying structure of a corpus of sequences. Because most naturally occurring sequences contain repetition and redundancy, grammars are often able to describe sequences efficiently.

### Algorithm

**Figure 1:**
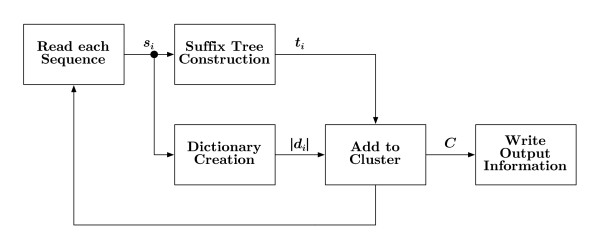
**Algorithm overview**. The algorithm operates on each sequence, *s_i_*, which is parsed into a suffix tree, *t_i_*, and dictionary, *d_i_*, for rapid distance comparison with other sequences. Each sequence is either added to an existing cluster, *c_j _*∈ *C*, or becomes the initial representative sequence in a new cluster, *c_k_*.

A general overview of the GramCluster algorithm is shown in Figure 1. The set of sequences, *S*, is regarded as input to the algorithm with *S *= {*s*_1_,...,*s_N_*}, where *s_i _*is the *i*th sequence and *i *∈ {1,..., *N*}. The goal of the algorithm is to partition *S *where each sequence is grouped with similar sequences from *S *such that all sequences within each resulting cluster are more similar to each other than sequences from other clusters. The final partition is represented by the set of clusters, *C *= {*c*_1_,..., *c_M_*}, where *c_j _*is the *j*th cluster and *j *∈ {1,..., *M*}. The algorithm initially generates a suffix tree, *t_i_*, and grammar dictionary, *d_i_*, associated with each sequence, *s_i_*. For each sequence, *s_i_*, these data structures are used to determine if an existing cluster contains sufficiently similar sequences to *s_i _*or if a new cluster needs to be created. If a cluster, *c_j _*∈ *C*, already exists with similar sequences, the sequence *s_i _*is added to *c_j_*. However, if no cluster contains similar sequences, a new cluster containing only *s_i _*is added to *C*. This clustering continues for all sequences in *S*. The algorithm is described in more detail below with reference to the various blocks in Figure 1.

#### Dictionary Creation

One of the core processes of the clustering algorithm is the formation of a distance estimate between an unprocessed sequence, *s_i_*, and each cluster, *c_j_*, already in the partition, *C*. To this end, one sequence, called the representative sequence, is used to represent all other sequences within each cluster. The distance between *s_i _*and $s_{r_{j}}\in c_{j}$, where $s_{r_{j}}$ represents *c_j_*, is used to determine if *s_i _*should be added to *c_j_*.

Each sequence, *s_i_*, is compared with, at most, the set of representative sequences, $\left\{s_{r_{j}}|s_{r_{j}}\;\text{represents}\;c_{j}\in C\right\}$, to discover the correct cluster for *s_i_*.

**Figure 2:**
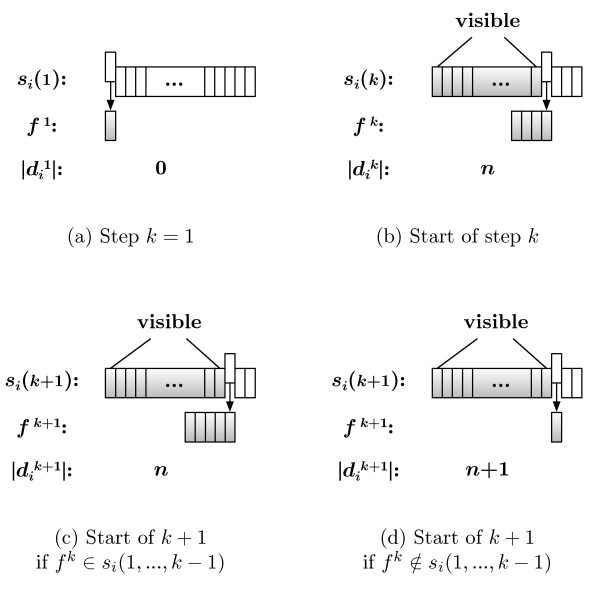
**Dictionary creation steps**. Determining the order of the LZ dictionary, |*d_i_|*, for sequence *s_i_*. (a) The initial step in which the initial fragment, *f*^1^, is set to the first letter, *s_i_*(1), of the sequence. (b) The start of the *k*th step in which the *k*th letter, *s_i_*(*k*), is appended to the current fragment, *f^k^*. After the first *k - *1 letters of *s_i _*are scanned for the occurrence of the fragment, *f_k_*, the two possible outcomes are (c) the fragment is reproducible with combinations of existing rules, or (d) the fragment is unique up to this point in the sequence, and so a new grammar rule is added to the dictionary and the fragment is reset.

The distance metric relies on the structural rules necessarily present in all information-containing sequences. GramCluster uses the grammar estimation method based on Lempel-Ziv (LZ) parsing [10,12,14] as used in [15] for language-phylogeny inference, in [16] for phylogeny reconstruction, and in [17] to construct a guide tree for multiple sequence alignment. A similar grammar-based distance is also the focus of [18] which analyzes the quality of the distance metric as a function of the length of the sequences. The primary aspects of LZ dictionary creation are shown in Figure 2 where a set of grammar rules for each sequence is calculated. Initially, the dictionary, $d_{i}^{1}=\emptyset$, is empty, a fragment, *f*^1 ^= *s_i_*(1), is set to the first residue of the corresponding sequence, and only the first element, *s_i_*(1), is visible to the algorithm. At the *k*th iteration of the procedure, the *k*th residue is appended to the fragment resulting from the (*k - *1)th step; and the visible sequence is checked. If *f^k ^*∉ *s_i_*(1,...,*k - *1), then *f^k ^*is considered a new rule and so added to the dictionary, $d_{i}^{k}=d_{i}^{k-1}\int \{f^{k}\}$; and the fragment is reset, $f^{k}=\emptyset$. However, if *f^k ^*∈ *s_i_*(1,...,*k - *1), then the current dictionary contains enough rules to reproduce the current fragment, i.e., $d_{i}^{k}=d_{i}^{k-1}$. In either case, the iteration completes by appending the *k*th residue to the visible sequence.

This procedure continues until the visible sequence is equal to the entire sequence, at which time the size of the dictionary, |*d_i_| *is determined for use in the metric calculation. The correlation of the LZ-based distance with phylogenetic distance was exploited in [16] to obtain phylogenies for a set of mammalian species using complete mitochondrial DNA and for the superfamily *Cavioidea *using exon#10 of the growth hormone receptor (GHR) gene, the transthyretin (TTH) gene, and the 12 S rRNA gene. In [19], the same distance metric was used to obtain phylogenies for fungal species using the cytochrome b gene and internal transcribed spacer regions of the rDNA gene complex.

#### Suffix Tree Construction

**Figure 3:**
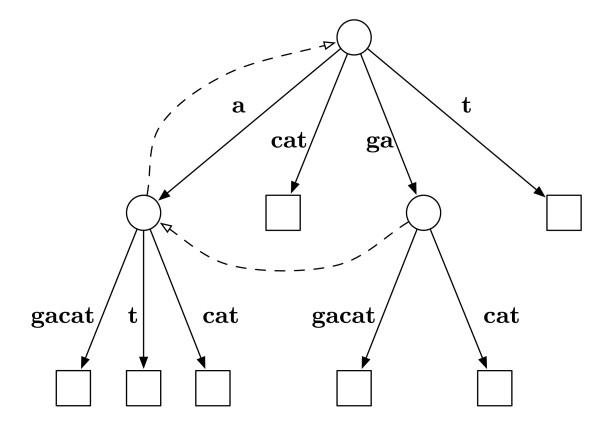
**Example suffix tree diagram**. Completed suffix tree diagram of the string "gagacat." Tracing a path from root to leaf along a solid line results in a suffix of the string. The dashed lines indicate suffix links that are useful during the creation of the suffix tree.

As shown in Figure 1 the algorithm also constructs a suffix tree for the sequence. Suffix trees are data structures designed to contain all *L *suffix substrings of a length-*L *sequence [20-22]. For example, a suffix tree for the sequence "gagacat" is schematically shown in Figure 3. All seven suffixes {gagacat, agacat, gacat, acat, cat, at, t} are found by tracing a unique path from the root node to one of the seven leaf nodes along solid lines. One valuable use of suffix trees is searching for substrings which can be thought of as the preffix of a suffix. By using a suffix tree, a length-*L *sequence can be completely scanned for a length-*F *fragment in O(*F*) time as opposed to O (*L*) for a brute force search. Also depicted in Figure 3 are the dashed-line suffix links which are a fundamental feature for linear-time construction of the suffix tree [22].

A sequence, *s_i_*, can be converted into a suffix tree, *t_i_*, in linear time and then searched for substrings in linear time based on the fragment length. As will be shown, suffix tree sequence representation is important for reducing the time required for GramCluster to complete all necessary grammar-based comparisons.

#### Clustering

**Figure 4:**
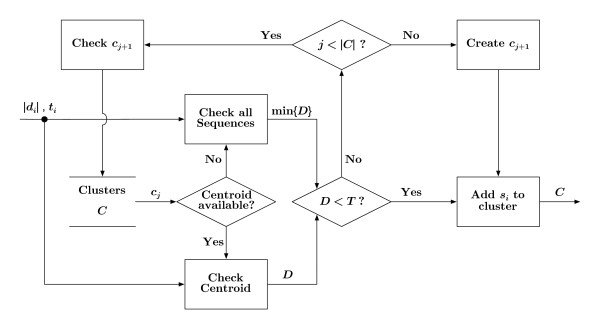
**Add to cluster**. A block diagram detailing the process by which sequence *s_i _*is added to a cluster, *c_j _*∈ *C*. A distance, *D*, is generated between *s_i _*and the representative of *c_j_*. If *D *is below a user-specified threshold, *T*, then *s_i _*is added to *c_j_*, otherwise the next cluster, *c*_*j*+1_, is checked. If no cluster is identified as suitable for *s_i_*, a new cluster containing *s_i _*is created and added to *C*.

The final component of the algorithm depicted in Figure 1 is represented by the block labeled, "Add to Cluster." The procedure for adding a sequence to a cluster is shown in greater detail in Figure 4. The algorithm checks each cluster, *c_j _*∈ *C*, until a cluster is found where the distance between the representative sequence, $s_{r_{j}}$ and *s_i_*,$D_{j}=\text{dist}(s_{i},s_{r})$ is less than a user-defined threshold, *T*. Once this condition is met, the cluster is updated, *c_j _*= *c_j _*∪ {*s_i_*}; and processing in this block terminates. If no clusters meet the condition of *D < T*, a new cluster is created with *s_i _*as its first member.

The following sections describe the cluster data structure, the representative sequence selection method, and the grammar-based distance calculation.

#### Cluster Data Structure

In order to follow the cluster classification process, it is helpful to understand the data structure used to represent each cluster. In particular, every cluster uses a list of suffix trees, *t_i_*, and dictionary sizes, |*d_i_*|, to identify its set of sequences. The remaining components contained in the data structure are used to determine and specify the representative sequence, $s_{r_{j}}$, of the cluster, *c_j_*. A good selection for $s_{r_{j}}$ is a sequence that appears grammatically similar to all other sequences within the cluster. This implies the need to estimate the grammar-based distance between all sequences of the cluster, a computationally expensive task. To avoid this cost, GramCluster selects only a few specific sequences in the cluster, that we will call "basis sequences," to which all others are compared. The representative sequence, $s_{r_{j}}$, can be determined by considering the sets of relative distances between all sequences and each basis sequence. The centroid of the cluster is then defined as the vector containing the mean values of each set of relative distances. The sequence with relative distances nearest to the centroid is selected as $s_{r_{j}}$.

**Figure 5:**
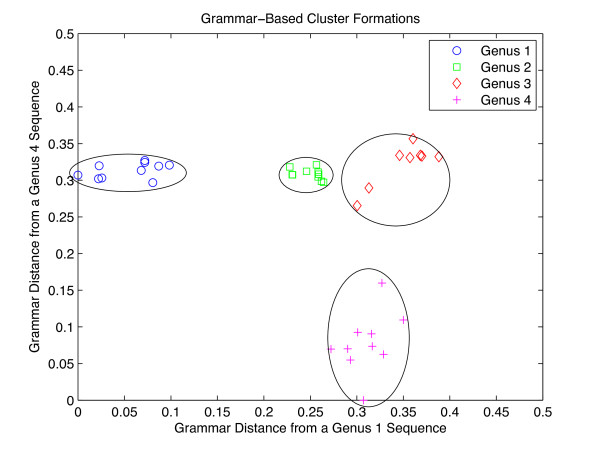
**Grammar-based cluster formations**. Forty sequences being processed via a vector quantizer. Each of the four genera is represented by ten sequences. Every sequence is grammatically compared to the same two sequences from within the set. The resulting pair of distances form two-dimensional vectors in a space. When considering the clusters in this space, the representative sequence of the cluster should be the sequence that is nearest the cluster center.

To see why this method is effective, consider that clustering is often performed in vector spaces where each element being classified is specified by a vector. The points spatially near each other are placed into the same cluster, and the representative is typically selected as the point that is closest to the center of the cluster. This idea is adapted in GramCluster, with an example depicted in Figure 5. The example in the figure contains forty sequences plotted in a two-dimensional space. Each dimension represents the grammar-based distance between the plotted sequence point and a basis sequence. The data set used in this example contained forty 16S rDNA sequences each from four genera (*Acetobacter*, *Achromobacter*, *Borrelia*, *Flavobacterium*). Of the two initially selected basis sequences, one came from *Acetobacter *and the second from *Flavobacterium*. Then, the pair of distances between each sequence and the basis sequences was computed and plotted. As can be seen from the plot, the sequences group into clusters which correspond to their genus. Note that the basis sequences are not orthogonal; however, use is made of the fact that the grammar-based distances tend to obey the transitive property such that if

$$\begin{array}{l} D_{b}=\text{dist}(s_{a},s_{b})\\ D_{c}=\text{dist}(s_{a},s_{c}) \end{array}$$

and if *D_b _*is close to *D_c_*, then *s_b _*and *s_c _*tend to be grammatically similar to each other. The example in Figure 5 demonstrates this by the use of basis sequences from *Acetobacter *(genus one) and *Flavobacterium *(genus four). One would expect that comparing all sequences to one sequence would provide separation between the sequences from the same genus as the basis sequence and the rest. However, sequences from the other genera also form into clusters as a result of sequences being compared to a single basis sequence. In our example, all forty sequences are compared to just two sequences; and four clear clusters appear. The method presented here for building vectors of distances relative to basis sequences is similar to the concept of embedding presented in [5]. The work of [5] details an algorithm called mBed that operates on a set of sequences to generate a distance matrix representing a phylogenetic guide tree, a process that is closely related to the data clustering problem presented here. The mBed algorithm selects a subset of *t *seed reference sequences that are not close together relative to a distance metric. Then each sequence has a *t*-dimensional vector associated with it where each coordinate value is the distance between the sequence and the respective reference sequence. The distance used in [5] was selected to be the *k*-tuple distance measure of [23] and implemented in ClustalW [24]. The basis sequence concept used in this work is similar, with the grammar-based distance metric replacing the *k*-tuple distance measure being the primary difference. Additionally, a single reference subset is used in [5] to build all vectors. The algorithm presented here creates vectors for each sequence contained in a cluster relative to basis sequences also sampled from the same cluster.

#### Representative Sequence Selection

As shown in Figure 4 the clustering process begins by comparing sequence *s_i _*to the representative sequence of cluster *c_j _*∈ *C*. For clusters containing many sequences, a representative sequence is determined using the basis sequence method described above. In this case, only the representative sequence, $s_{r_{j}}$, is compared to *s_i_*

$$D=\text{dist}(s_{i},s_{r_{j}}).$$

However, the progressive addition of sequences to clusters means there are clusters containing only a few sequences. These clusters do not contain a large enough sample set to yield a reliable representative. Thus, until a cluster is large enough, all sequences are considered representative and compared to *s_i_*

$$D_{k}=\text{dist}(s_{i},s_{k})\;\forall s_{k}\in c_{j}.$$

The minimum distance, $\underset{k}{\min}\{D_{k}\}$, is used as the classification metric.

#### Grammar-Based Distance Calculation

The distance metric used in GramCluster is a modified form of the grammar-based distance metric introduced in [16,18] and used in [17].

The original distance metric is computed by concatenating the two sequences being compared into a single sequence and then performing the operations detailed in Figure 2. Formally, consider the process of comparing sequences *s_m _*and *s_n_*. Initially, the dictionary, $d_{m,n}^{1}=d_{m}$, is set to that of sequence *s_m_*, a fragment, *f*^1 ^= *s_n_*(1), is set to the first residue of the *n*th sequence, and the visible sequence is all of *s_m_*.

The algorithm operates as described previously, resulting in a new dictionary size, |*d*_*m*,*n*_|. When complete, more grammatically similar sequences will have a new dictionary size with fewer entries as compared to sequences that are less grammatically similar. Therefore, the size of the new dictionary, |*d*_*m*,*n*_|, will be close to the size of the original dictionary, |*d_m_|*. The distance between the sequences is estimated using the dictionary sizes, in particular

(1) $$D=\{\begin{array}{cc} \frac{\left|d_{m,n}|-|d_{m}\right|}{d_{m}}\times \frac{\left|s_{m}\right|}{\left|s_{n}\right|} & \text{if}|s_{m}|\;>\;|s_{n}|,\\ \frac{\left|d_{n,m}|-|d_{n}\right|}{\left|d_{n}\right|}\times \frac{\left|s_{n}\right|}{\left|s_{m}\right|} & \text{if}|s_{m}|\;\leq \;|s_{n}|. \end{array}$$

This particular metric accounts for differences in sequence lengths and normalizes accordingly. Smaller values of *D *indicate a stronger similarity. Intuitively, sequences with a similar grammar should be clustered with each other.

While this grammar-based distance metric works well, it requires that the extended sequence be rescanned for every residue in the second sequence. This means that *s_m _*will be rescanned completely for every character in *s_n_*. This process is repeated as many times as the number of sequences compared to *s_m_*. As a result, approximately 75% of the computation is devoted to string searching and concatenation. To improve the execution time, we introduce two significant modifications described below.

#### Fragment Markers

**Figure 6:**
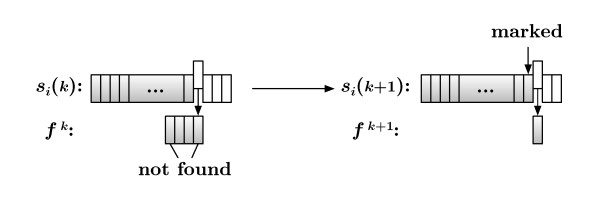
**Fragment markers**. One of the implementation optimizations is marking locations in the sequences where fragments are not found in the visible sequence. Doing so eliminates the need to rescan sequences during the distance calculation for fragments that are already known to be found within the original sequence.

The original distance calculation would simply repeat the process depicted in Figure 2 on the concatenation of two sequences being compared. Thus, for the *k*th character in the second sequence, the first sequence is completely scanned along with the initial *k *- 1 portion of the second sequence. However, this is quite unnecessary since many fragments formed from the second sequence were already found in the second sequence during the initial scan. Formally, consider sequences *s_m _*and *s_n _*which have already had their own dictionaries created in a previous step. Now suppose the concatenated sequence *s*_*m*·*n *_is being processed for the *k*th character in *s_n_*, at which point there is a nonempty fragment, *f^k^*. The process begins with the fragment completely composed of consecutive letters from *s_n_*, which means that this fragment has already been created once before when *s_n _*was processed by itself. As long as *f^k ^*was previously found within *s_n_*(1,..., *k - *1), there will be no new information gained by scanning *s_m·n_*(1,..., *|s_m_| *+ *k - *1), because it is certain to be there since *s_n_*(1,..., *k - *1) ⊂ *s_m·n_*(1,..., *|s_m_| *+ *k - *1). So, there is no need to scan for fragments that have been previously found during any distance calculation. The inverse statement is also true: fragments not previously found do need to be scanned for during a distance calculation. This is implemented as shown in Figure 6 in which fragment *f^k ^*∉ *= s_i_*(1,..., *k - *1), so *k *is added to a list of marked fragment indices.

The same distance metric given by (1) is used, but there is no longer a need to perform string concatenation; and only the first string is scanned for the marked fragments from the second string. Formally, consider the process of comparing sequences *s_m _*and *s_n_*. Initially, the dictionary, $d_{m,n}^{1}=d_{m}$, is set to that of sequence *s_m_*, a fragment, *f *^marked(1)^, is set to the first marked substring of the *n*th sequence, and the visible sequence is always just *s_m_*. The algorithm simply scans *s_m _*for an occurrence of the fragment and adds one to the dictionary if the fragment is not found. Either way, the fragment is updated to the next marked substring of *s_n_*; and *s_m _*is scanned again. This continues for all marked fragments from *s_n _*resulting in a new dictionary size, | *d_m,n_*|. This fragment marking process significantly reduces the total number of substring searches performed, as well as the character concatenations that would be otherwise required.

The second optimization involves a time-efficient method of searching a string for a substring of characters, a very relevant problem for suffix trees.

#### Suffix Tree Searches

As stated previously, a length-*L *sequence stored in a suffix tree data structure can be completely scanned for a length-*F *fragment in O(*F*) time. To see why this is true, consider the simple example depicted in Figure 3. Every suffix is represented in the data structure as a unique path beginning at the root node and traversing along a solid line to a leaf node. Any substring occurring in this string has to be the start of a suffix, so searching for a substring amounts to finding a suffix that begins with the substring. Consider searching "gagacat" for the substring fragment "gac" which is present in the string. The first step is to find a branch beginning with "g" leaving the root, which is found as the third entry in the data structure.

Following the branch to the internal node indicates that all suffixes in this tree that begin with "g" are always followed by an "a," which is also true of the fragment. At the internal node, the next step is to search for any branch that begins with "c," which is found as the second entry in the data structure, concluding the search. Next, consider searching for the substring fragment "gact," which follows the previous search to the internal node and includes identifying the branch beginning with "c." The final step is looking at the subsequent character along the branch, which is "a," and does not match. This search finishes having determined that "gact" is not a substring of "gagacat." The use of the suffix tree in this context means that the time necessary for identifying whether previously marked fragments from sequence *s_n _*are present in sequence *s_m _*is O(*F *).

#### Algorithm Complexity

The algorithm complexity of GramCluster may be broken into three pieces, beginning with the generation of each sequence grammar dictionary, *d_i _*for *i *∈ {1, *..., N*}, where *N *is the number of sequences. Suppose the average sequence length is *L*, then each *d_i _*results in complexity O (*L*), so all dictionaries are generated with complexity O (*LN*). Next, each suffix tree, *t_i_*, has a complexity O (*L*^2^), so all sequences are converted into trees with complexity O(*L*^2^*N*). Finally, suppose the average number of clusters is *M*. As an upper bound, all clusters are scanned until each sequence is classified and each scanning process has complexity O(*L*). The result is a total scanning complexity of O(*LMN*). Thus, the entire time complexity for GramCluster is O(*LN *+ *L*^2^*N *+ *LMN*), which simplifies to O(*L*^2^*N *+ *LMN*).

Regarding the memory complexity of GramCluster and continuing with *N *as the number of sequences, suppose the average sequence header length in the FASTA file is *H*. Because every header line is stored for subsequent file output, this memory complexity is O(*HN*). As before, if the average sequence length is *L*, then each sequence is stored in O(*L*). The worst-case memory usage for the clusters themselves occurs if every cluster created has an incomplete set of basis sequences. In this case, each cluster has a memory complexity of O(*C *+ *B *+ *BC *+ *LC*) where *C *is the number of sequences held within the cluster and *B *is the number of basis sequences per cluster. Because there are *N *sequences stored in memory during this worst-case scenario, a final upper bound on the memory complexity is O((*H *+ *B *+ *L*)*N*) in which the most significant component has a memory complexity of O(*LN*).

### Testing

We performed several clustering experiments to validate the proposed algorithm, GramCluster version 1.3 (see Additional File 1). The training procedures for obtaining the default parameters are described in the Methods section. In particular, we used GramCluster to cluster sets of 16S rDNA sequences. As detailed in the Methods section, the resulting clusters were analyzed for correctness whereby the genus of each sequence was compared to that of all other sequences in the data set. Correct classification is considered when sequences belonging to the same genus fall into the same cluster. Likewise, incorrect classification occurs when sequences belonging to different genera are placed into the same cluster. Each output set was analyzed using several statistical quality metrics described in the Methods section. For comparison, CD-HIT-EST (no version given, archive created on 4/27/2009) [6] and UCLUST version 3.0.617 [8] were also used to cluster the same 16S rDNA sequences and analyzed using the same quality metrics.

#### Experiments with Moderate-Sized Data Set

**Figure 7:**
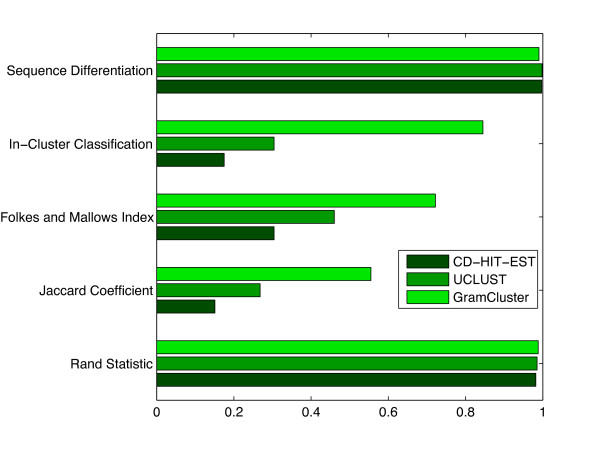
**Cluster metrics on moderate-sized data set**. Cluster metrics for each algorithm operating on 74,709 16S rDNA sequences from 2,255 different genera.

The proposed algorithm was evaluated using the Folkes and Mallows Index, the Jaccard Coefficient, and Rand Statistic measures [25], along with in-cluster classification and sequence differentiation percentages, all defined in the Methods section. The results for GramCluster, CD-HIT-EST, and UCLUST are presented in Figure 7.

Results indicate that CD-HIT-EST achieved 17.5% in-cluster classification and 99.7% sequence differentiation out of the 2,050 total clusters determined. That is, for sequences that were supposed to be in the same cluster, CD-HIT-EST placed them together 17.5% of the time; and for sequences that were not supposed to be in the same cluster, it correctly kept them in different clusters 99.7% of the time. Improved results for UCLUST show 30.4% and 99.8% in-cluster classification and sequence differentiation out of the 1,680 total clusters determined. By comparison, GramCluster achieved 84.5% in-cluster classification and 99.0% sequence differentiation out of the 2,447 total clusters identified. Clearly, GramCluster provides a significant improvement in clustering sequences correctly. This improvement can be further observed using common statistical measures for evaluating the performance of clustering algorithms [25] described in the Methods section. These measures are shown for GramCluster, CD-HIT-EST, and UCLUST operating on a set of 74,709 16S rDNA genes obtained from 2,255 different genera. The Jaccard Coefficient and Folkes and Mallows Index exceed those of CD-HIT-EST four-fold and over two-fold, respectively. The CPU execution time of GramCluster (1342 seconds) is on the same order as that of CD-HIT-EST (8277 seconds), which is considered ultra-fast [26]. The UCLUST CPU execution time (89 seconds) is much faster than GramCluster, however its quality metrics fall significantly short of those provided by GramCluster.

#### Experiments with Large Data Set

**Figure 8:**
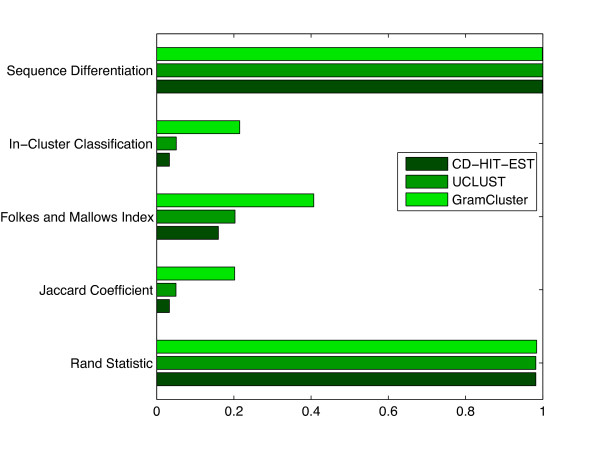
**Cluster metrics on large data set**. Cluster metrics for each algorithm operating on 1,045,926 16S rDNA sequences from 2,255 different genera.

In order to simulate the application of clustering a large set of unknown fragments that typically result from 454 pyrosequencing, the previous FASTA file was modified such that every sequence was reduced to only the first 200 bases and then repeated 14 times for a total of 1,045,926 sequences from 2,255 genera. Figure 8 contains data covering the same categories as in the previous experiment. CD-HIT-EST achieved only 3.3% in-cluster classification and 99.9% sequence differentiation of the 11,758 clusters found. So, for sequences that were supposed to be in the same cluster, CD-HIT-EST placed them together 3.3% of the time; and for sequences that were not supposed to be in the same cluster, it correctly kept them in different clusters 99.98% of the time. As in the previous experiment, results for UCLUST show 5.1% and 99.9% in-cluster classification and sequence differentiation out of the 10,686 total clusters determined. By comparison, GramCluster achieved 21.5% and 99.9% out of the 5,917 clusters identified. GramCluster continues to show a significant improvement in terms of clustering sequences correctly with each other. This improvement can be seen further with the higher statistical measures, especially in the Jaccard Coefficient and Folkes and Mallows Index which are over six and two times those of CD-HIT-EST. Perhaps most interestingly, GramCluster identified a more accurate number of clusters at 5,917, even though the length of the sequences was significantly reduced, while both CD-HIT-EST and UCLUST reported identifying over 10,000 clusters.

**Figure 9:**
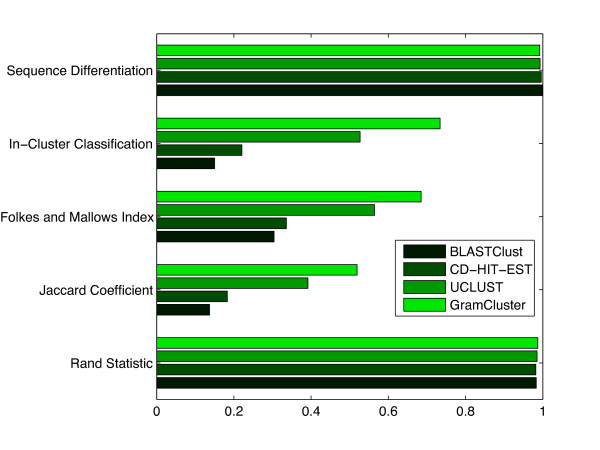
**Cluster metrics on small data set**. Cluster metrics for each algorithm operating on 7,470 16S rDNA sequences from 898 different genera.

We also tested BlastClust [9] on 16S sequences. The program was too slow for classifying the original set of 74,709 sequences so we tested it using only 10% of the sequences. The results are shown in Figure 9. As can be seen, the results of CD-HIT-EST, UCLUST, and GramCluster all tend to match those of Figure 7. As can be seen in Figure 9 BlastClust resulted in lower statistical metric scores in all categories, a high number of clusters compared to the number of genera. It is clear that the exclusion of BlastClust from the other experiments due to its inability to operate on the size of the input data set has not diminished the results.

#### Varying Command Line Options

Next, we consider the effect of varying the command line options primarily responsible for affecting the resulting data set partition. We ran two additional clustering experiments on the original set of sequences with GramCluster and UCLUST. The GramCluster experiments had both grammar-based distance thresholds altered from the default setting of 0.13 to 0.15 and 0.11. Similarly, the UCLUST experiments had the identity threshold altered from the default setting of 90% to 85% and 95%.

**Figure 10:**
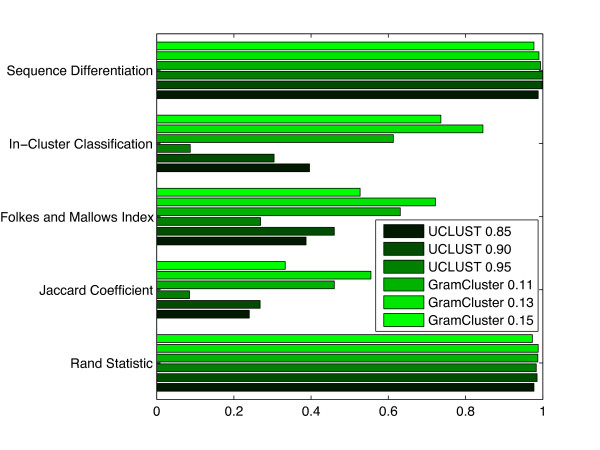
**Cluster metrics by varying thresholds**. Cluster metrics for GramCluster and UCLUST operating on 74,709 16S rDNA sequences from 2,255 different genera. The grammar-based distance thresholds were both set to 0.11, 0.13, and 0.15 for GramCluster. The identity threshold was set to 85%, 90%, and 95% for UCLUST.

Figure 10 contains data covering the same categories as in the previous experiments. As the grammar-based distance threshold increased, sequences that were increasingly dissimilar were clustered together resulting in fewer clusters and poorer metrics. This same trend occurred with UCLUST as the identity threshold was relaxed by reducing it. Likewise, when the grammar-based distance threshold was reduced, sequences with an appropriately smaller distance clustered together. Similar behavior occurred when the UCLUST identity threshold was increased. In general, the default parameters for both programs seem to provide the best clustering of genus based on overall comparison of the metrics in Figure 10.

#### Experiments Clustering on Species

The final experiment operated on the original set of sequences, but the partitioning was based on the sequence species instead of their genus.

**Figure 11:**
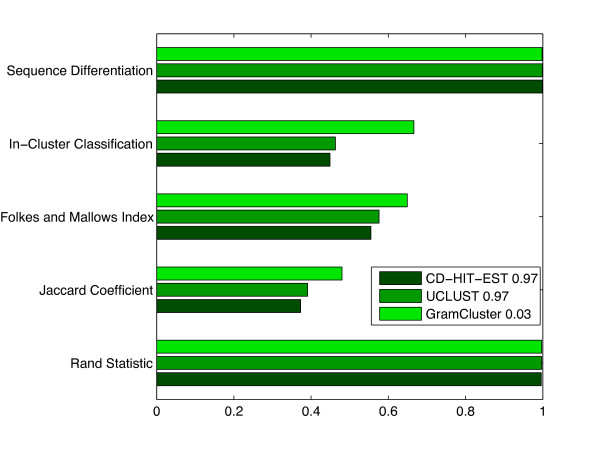
**Cluster metrics by species**. Cluster metrics for each algorithm operating on 29,566 16S rDNA sequences from 5,472 different species.

Figure 11 contains data covering the same categories as in the previous experiments. In order to achieve the metrics in Figure 11 based on sequence species, it was necessary to modify the threshold of each clustering program. The UCLUST and CD-HIT-EST percent identity parameter was adjusted upward to require a higher sequence similarity before clustering sequences together. The best overall metric scores based on sequence species occurred at 97% identity for each algorithm. In contrast, the grammar-based distance thresholds in GramCluster had to be lowered to restrict the distance between sequences before classifying them together. The threshold of 0.03 caused the best overall metrics due to sequence species. The results presented in Figure 11 show a similar trend to those of the first experiment in Figure 7. The results from all experiments show viable promise of the proposed algorithm, especially when clustering numerous sequences such as in datasets produced by high-throughput sequencing applications.

## Conclusions

The primary goal of this work was to introduce a computationally efficient clustering algorithm which can be used for clustering large datasets with high accuracy. The algorithm introduced was validated against a specific class of datasets containing 16S rDNA sequences but was designed to cluster any set of RNA, DNA, or protein sequences. The grammar-based distance work introduced in [16,18] and previously used in [17] was modified to generate an estimation of the proper classification in which sequences are to be grouped. Results from clusters generated were presented in an attempt to study the overall quality of the resultant classifications as well as the computation time necessary to achieve the outputs. Accurate clustering of large numbers of biological sequences in an efficient amount of time is an important and challenging problem with a wide spectrum of applications. In this work, we adapted existing ideas in a novel way and introduced significant improvements. The proposed algorithm achieved higher-quality clusters compared to existing methods while operating at similar, high-speed execution times.

## Methods

### Experiments

All results presented in the Testing section were generated by compiling and executing the respective clustering programs on the same computer, specifically an Apple MacBook Pro with an Intel Core 2 Duo operating at 2.53 GHz with 4 Gb of system memory and a 3 Mb L2 cache. In the case of UCLUST, the binary was downloaded from the author's website. The experiments were conducted using various versions of FASTA files containing 74,709 16S rDNA sequences from 7,043 different species of 2,255 genera obtained from the Ribosomal Database Project http://rdp.cme.msu.edu. For example, the second set of experiments involved a processed version of the FASTA file to simulate the application of clustering a large set of unknown fragments that typically result from high-throughput sequencing technologies, such as 454 pyrosequencing. In particular, every sequence was reduced to only the first 200 bases; and then the entire file was repeated 14 times for a total of 1,045,926 sequences from 2,255 genera.

In each file, the header line of each sequence was replaced by an integer number associated with that sequence's genus. In this way, the resulting clusters could be validated for quality by comparing the header integers with all other entries. In particular, we used three statistical measures, identified in [25], to assess the quality of resulting clusters, including the Rand Statistic, the Jaccard Coefficient, and the Folkes and Mallows Index. In all cases, a count was created based on the pair-wise comparison of each element with all other elements being clustered. When two elements were compared, and they fell into one of four possible categories: 1) the pair should be in the same cluster and they are in the same cluster (*SS*), 2) the pair should be in different clusters but they are in the same cluster (*DS*), 3) the pair should be in the same cluster but they are in different clusters (*SD*), and 4) the pair should be in different clusters and they are in different clusters (*DD*). The goal of a clustering algorithm is to obtain maximal values for *SS *and *DD *and minimal values for *DS *and *SD*. The three metrics all operate on combinations of these counts in order to provide an indication as to the quality of actual clustering versus ideal clustering, as follows:

$$\begin{array}{l} s_{\text{RS}}=(SS+DD)/(SS+DS+SD+DD)\\ s_{\text{JC}}=SS/(SS+DS+SD)\\ s_{\text{FMI}}=SS/\sqrt{\left(SS+DS)(SS+SD\right)}. \end{array}$$

Notice all metrics are bounded between 0 and 1, with 0 being a poor clustering score and 1 a perfect clustering score. Additionally, the in-cluster classification and sequence differentiation percentages

$$\begin{array}{l} s_{\text{in}}=SS/(SS+SD)\\ s_{\text{diff}}=DD/(DS+DD) \end{array}$$

are provided. Given all sequence pair comparisons, the total number that implies a pair of sequences belong to the same genus is (*SS *+ *SD*). Of that total, only *SS *pairs were actually classified into the same cluster. Thus, the in-cluster classification is the percent of sequence-to-sequence pairs that have correctly clustered sequences together out of all that should be clustered together. Similarly, the total number of sequence pair comparisons that imply two sequences do not belong to the same genus is (*DS *+ *DD*). Out of the total, only *DD *pairs were correctly separated into different clusters. The sequence differentiation used here was the percent of sequence pair comparisons that have correctly classified sequences apart out of the total that should not be clustered together.

We repeated the first two experiments in the Testing section using two different random permutations of the FASTA file (results not shown). All programs produced very similar results, thereby demonstrating that the order in which sequences are input to the algorithms does not affect the resulting clusters. In order to identify the best set of default parameters for the GramCluster implementation, we used two different training methods. In the first method, we randomly selected 10% of the sequences for training while the remaining 90% were used for testing. In the second method, we randomly divided the genera into two sets, one containing about 10% of the sequences and the other containing 90% of the sequences. The smaller set was again used as a training set to obtain the parameters for the algorithm. The default parameters ended up being the same as those found in the first training experiment. In particular, a grammar-based threshold of 0.13 was found to produce the best overall clustering metrics based on genera.

We applied the same training methods to identify the best thresholds for GramCluster when clustering based on species. In this case, the best overall clustering metrics based on species occurred when the grammar-based threshold of 0.03 was applied.

### Command Line Options

The following list details the user-definable command line options available in the current GramCluster implementation.

1. -B <value> Specify the full basis amount. The value specified in this option represents the number of nonidentical sequences added to a cluster before a centroid sequence is determined. If this option is not specified, the default value is 4 sequences.

2. -b <value> Specify the grammar distance identical threshold. The value specified in this option represents the grammar-based distance threshold for two sequences to be consider grammatically identical. When a new sequence is added to a cluster, it has a distance less than one of the thresholds (specified by -C or -G). In the event that two sequences are very similar (or identical), this threshold prevents the new sequence from becoming a basis sequence. If this option is not specified, the default value is 0.01.

3. -C <value> Specify the grammar distance-to-centroid maximum threshold. The value specified in this option represents the grammar-based distance threshold to the centroid sequence. If a distance calculated between a new sequence and the centroid sequence is less than this value, then the new sequence is added to the cluster. If this option is not specified, the default value is 0.13.

4. -G <value> Specify the grammar distance maximum threshold. The value specified in this option represents the grammar-based distance threshold to all basis sequences for clusters that do not have a centroid already determined. If a distance calculated between a new sequence and any basis sequence is less than this value, then the new sequence is added to the cluster. If this option is not specified, the default value is 0.13.

5. -c Turn on complete cluster searching. This causes the algorithm to scan every cluster for the lowest distance before adding it. The default is greedy cluster searching, which causes sequences to be added to the first cluster presenting a distance lower than the specified thresholds.

6. -R Turn on reverse complement checking. This causes GramCluster to check both the input sequence as well as its reverse complement against each cluster representative. The lowest resulting distance is used for classification.

Note that the -C and -G options specify thresholds that function similar to the identity percentage thresholds used by other clustering programs, such as CD-HIT-EST and UCLUST. However, the thresholds function just the opposite, whereby sequences are only added if their grammar-based distance is calculated as a value below the threshold value. In contrast, the identity percent thresholds of CD-HIT-EST and UCLUST require sequences to have a metric score higher than the threshold before they are added to the respective cluster.

### Availability

The source code for the current version of GramCluster may be downloaded from http://bioinfo.unl.edu/.

## Authors' contributions

DJR conceived the idea of using fragment markers and suffix trees along with the vector quantization paradigm, implemented the entire algorithm, performed all evaluations, and drafted the initial manuscript. SFW implemented early versions of the clustering algorithm based on modifications to the distance matrix method employed in GramAlign and provided Python script support for manipulating the FASTA test files. AKB posed the initial problem of a clustering application. KS collaborated with AKB, SFW, and DJR in the development of the algorithm and preparing the final manuscript. All authors read and approved the final manuscript.

## Supplementary Material

Additional file 1 **An executable may be generated by unzipping this file and using an ANSI C compiler to build the code**. Click here for file
